# Supplementary material for: An open-source framework for large-scale, flexible evaluation of biomedical text mining systems
Source: J Biomed Discov Collab. 2008 Jan 29;3:1. doi: 10.1186/1747-5333-3-1 (PMC2276192; doi:10.1186/1747-5333-3-1)
Supplement: Additional file 1 — Evaluation platform source code. The additional file contains the software evaluation framework discussed in this paper. The most current release can be downloaded from SourceForge.net [1]. See the accompanying README file for instructions on installation and use. Also included in the distribution is a collection of UIMA wrappers for some commonly used BioNLP tools and annotated corpora. [file 1747-5333-3-1-S1.zip › bionlp_uima_repository-1.0.1/docs/api/src-html/edu/uchsc/ccp/uima/ae/nlp/ei/gene/AbGene_AE.html]

```
001    /*
002     * Created on Sep 8, 2005
003     *
004     * TODO To change the template for this generated file go to
005     * Window - Preferences - Java - Code Style - Code Templates
006     */
007    package edu.uchsc.ccp.uima.ae.nlp.ei.gene;
008    
009    import java.util.Iterator;
010    import java.util.List;
011    
012    import org.apache.uima.analysis_engine.ResultSpecification;
013    import org.apache.uima.analysis_engine.annotator.AnnotatorConfigurationException;
014    import org.apache.uima.analysis_engine.annotator.AnnotatorContext;
015    import org.apache.uima.analysis_engine.annotator.AnnotatorContextException;
016    import org.apache.uima.analysis_engine.annotator.AnnotatorInitializationException;
017    import org.apache.uima.analysis_engine.annotator.AnnotatorProcessException;
018    import org.apache.uima.analysis_engine.annotator.JTextAnnotator_ImplBase;
019    import org.apache.uima.jcas.JCas;
020    
021    import edu.uchsc.ccp.uima.annotation.CCPDocumentInformation;
022    import edu.uchsc.ccp.uima.util.UIMA_Util;
023    import edu.uchsc.ccp.util.external.abgene.AbGeneEISystem;
024    import edu.uchsc.ccp.util.nlp.annotation.AnnotationSet;
025    import edu.uchsc.ccp.util.nlp.annotation.TextAnnotation;
026    
027    /**
028     * @author williamb Takes longer time to run the first time.. not sure why
029     */
030    public class AbGene_AE extends JTextAnnotator_ImplBase {
031        private final boolean DEBUG = false;
032    
033        private AbGeneEISystem eiSystem;
034    
035        private String abgeneBaseDirectory;
036    
037        /**
038         * Name of configuration parameter that must be set to the path of the directory TnT will use for storing files generated in the gene
039         * annotation process.
040         */
041        public static final String PARAM_ABGENE_BASE_DIRECTORY = "AbGeneBaseDirectory";
042    
043        public void initialize(AnnotatorContext ac) throws AnnotatorInitializationException, AnnotatorConfigurationException {
044    
045            try {
046                abgeneBaseDirectory = (String) ac.getConfigParameterValue(PARAM_ABGENE_BASE_DIRECTORY);
047            } catch (AnnotatorContextException ace) {
048                ace.printStackTrace();
049                abgeneBaseDirectory = null;
050            }
051            System.err.println("Initializing AbGene EI System ...");
052            eiSystem = new AbGeneEISystem(abgeneBaseDirectory);
053            super.initialize(ac);
054        }
055    
056        /*
057         * (non-Javadoc)
058         * 
059         * @see com.ibm.uima.analysis_engine.annotator.JTextAnnotator#process(com.ibm.uima.jcas.impl.JCas,
060         *      com.ibm.uima.analysis_engine.ResultSpecification)
061         */
062        public void process(JCas jcas, ResultSpecification arg1) throws AnnotatorProcessException {
063    
064            /* get document text */
065            String docText = jcas.getDocumentText();
066    int documentCollectionID = -1;
067            Iterator docInfoIter = jcas.getJFSIndexRepository().getAnnotationIndex(CCPDocumentInformation.type).iterator();
068            String docID = "-1";
069            if (docInfoIter.hasNext()) {
070                CCPDocumentInformation srcDocInfo = (CCPDocumentInformation) docInfoIter.next();
071                docID = srcDocInfo.getDocumentID();
072                documentCollectionID = srcDocInfo.getDocumentCollectionID();
073            } else {
074                System.err.println("ERROR -- AbGeneAnalysisEngine: No CCPDocumentInformation associated with the JCas");
075            }
076    
077            
078         /* we standardize the documentid to be 123456789 because some document IDs (e.g. BioCreative) are tagged as genes and this messes things up. */
079            /* run the ei system */
080            List<TextAnnotation> proteinAnnotations = eiSystem.getEntitiesFromString(docText, "123456789");
081            
082            
083    
084            if (DEBUG) {
085                System.err.println("Using ABGENE on document: [" + docID + "] " + docText);
086                System.err.println("# Annotations Found: " + proteinAnnotations.size());
087                
088            }
089            
090            AnnotationSet aset = new AnnotationSet(new Integer(16),"Pre-tagged proteins and cell types","This annotation set encompasses annotations that were pre-tagged using automated methods.");
091            
092            /* Add -1 entrez_gene_id for each protein annotation */
093            for (TextAnnotation ta : proteinAnnotations) {
094                ta.setDocumentID(docID);
095                ta.setDocumentCollectionID(documentCollectionID);
096                ta.addAnnotationSet(aset);
097    //            ClassMention cm = ta.getClassMention();
098    //            /* make sure it is a protein */
099    //            if (cm.getMentionName().equals(ClassMentionTypes.PROTEIN)) {
100    //                SlotMention sm = cm.getSlotMentionByName(SlotMentionTypes.PROTEIN_ENTREZGENEID);
101    //                if (sm != null) {
102    //                    sm.setFirstSlotValue("-1");
103    //                } else {
104    //                    sm = new SlotMention();
105    //                    sm.setMentionName(SlotMentionTypes.PROTEIN_ENTREZGENEID);
106    //                    sm.setFirstSlotValue("-1");
107    //                    cm.addSlotMention(sm);
108    //                }
109    //            }
110            }
111            
112            
113            /* add protein annotations to the CAS */
114            UIMA_Util uimaUtil = new UIMA_Util();
115            uimaUtil.putTextAnnotationsIntoJCas(jcas, proteinAnnotations);
116    
117        }
118    }
```
